# Inflammatory Marker but Not Adipokine Predicts Mortality among Long-Term Hemodialysis Patients

**DOI:** 10.1155/2007/19891

**Published:** 2007-12-06

**Authors:** Yu-Che Tsai, Chien-Te Lee, Tiao-Lai Huang, Ben-Chung Cheng, Chien-Chun Kuo, Yih Su, Hwee-Yeong Ng, Chih-Chau Yang, Fong-Rong Chuang, Shang-Chih Liao

**Affiliations:** ^1^Division of Nephrology, Department of Internal Medicine, Chang-Gung Memorial Hospital, Kaohsiung Medical Center, Chang Gung University, College of Medicine, Kaohsiung Hsien 833, Taiwan; ^2^Department of Psychiatry, Chang-Gung Memorial Hospital, Kaohsiung Medical Center, Chang Gung University, College of Medicine, Kaohsiung Hsien 833, Taiwan; ^3^Department of International Trade, Cheng Shiu University, Kaohsiung Hsien 833, Taiwan

## Abstract

*Aims*: chronic inflammation contributes significantly to the morbidity and mortality of chronic hemodialysis patients. A recent research has shown that adipokines were associated with inflammation in these patients. We aim to investigate whether biomarkers of inflammation, adipokines, and clinical features can predict the outcome of hemodialysis patients. *Materials and methods*: we enrolled 181 hemodialysis patients (men: 97, mean age: 56.3±13.6) and analyzed predictors of long-term outcomes. *Results*: during the 3-year followup period, 41 patients died; the main causes of death were infection and cardiovascular disease. Elevated serum levels of hsCRP and albumin and advanced age were highly associated with death (all P<.001). Leptin and adiponectin levels were not significantly different between deceased patients and survivors. Cox-regression analysis indicated that age, diabetes, albumin level, and hsCRP were independent factors predicting mortality. *Conclusion*: the presence of underlying disease, advanced age, and markers of chronic inflammation is strongly related to survival rate in long-term hemodialysis patients.

## 1. INTRODUCTION

Patients with chronic renal disease have an elevated risk of cardiovascular disease. For example, a recent study reported that cardiovascular mortality in dialysis patients is 10 to 20 times higher than the general population [1]. Malnutrition and inflammation are often present in patients with chronic renal disease and are strongly associated with clinical outcome. Therefore, some researchers have used the terms “malnutrition-inflammation complex syndrome” or “malnutrition, inflammation, and atherosclerosis syndrome” to indicate the interplay of these conditions in dialysis patients [2, 3]. Among the markers of malnutrition that predict mortality in end stage renal failure (ESRF) patients, serum albumin is more closely associated to comorbidity and age than nutritional status *per se* [4]. Proinflammatory markers such as interleukin-6 (IL-6) and tumor necrosis factor alpha (TNF-α) induce hepatic synthesis of the acute phase reactant, CRP, which is a cardiovascular risk marker and an independent risk factor [5]. Recent studies have shown that inflammation plays a more important role than hypoalbuminemia in the pathogenesis of cardiovascular disease [6, 7].

The causes of inflammation among dialysis patients are complex and multifarious. A recent study indicated that fat tissue secrets numerous adipokines, such as leptin and adoponectin that contribute to systemic inflammation in dialysis patients [8]. In the present study, we aim to examine whether demographic data, biomarker of inflammation, nutritional status, and adipokine can predict the outcome of long-term hemodialysis patients.

## 2. SUBJECTS AND METHODS

### 2.1. Subjects

In August 2002, we enrolled a cohort of 181 Taiwanese with ESRF (97 men and 84 women) who had received regular hemodialysis three times a week for at least six months. We followed the patients for 36 months. The underlying causes of renal failure were chronic glomerulonephritis (N = 80), diabetes mellitus (N = 54), hypertension (N = 7), polycystic kidney disease (N = 5), interstitial nephritis (N = 5), systemic lupus erythematous (SLE, N = 4), obstructive uropathy (N = 1), and unknown etiology (N = 25). All four patients with SLE were free of disease activity as determined by clinical judgment and serological markers. None of the patients suffered from acute infection, inflammation, or viral hepatitis at the time of enrollment. Patients with abnormal liver function results or leucocytosis were excluded from this study to ensure they were free of infection. All patients received hemodialysis 4 hours per session and three sessions per week. We prospectively followed these patients for 36 months and recorded the survival rate and causes of mortality.

At the initiation of the study, we recorded patient demographics (age, gender, duration of dialysis therapy, and body mass index, BMI) and the presence of comorbid conditions such as diabetes mellitus and hypertension. The agents used for blood pressure control were reviewed and recorded in hypertensives. The duration of hemodialysis is defined as the number of months from commencement of regular hemodialysis until enrollment in the present study. We assessed adequacy of dialysis by $\text{K}\text{T}/{\text{V}}_{\text{urea}}$ and urea reduction ratio (URR). We used the urea kinetic model of Daugirdas to determine the $\text{K}\text{T}/{\text{V}}_{\text{urea}}$. Normalized protein catabolic rate (n-PCR, g/kg/day) represents nutritional intake. This study had been approved by the Institutional Review Boards and Ethics Committees and all patients were fully informed of their participation in this study.

### 2.2. Laboratory measurements

We collected fasting blood samples at midweek immediately prior to the start of hemodialysis via an arterial line and stored samples at −80°C. Biochemical data (serum albumin, total cholesterol, triglyceride, and hemoglobin) and inflammatory markers (high-sensitivity CRP (hsCRP) interleukin-6, IL-6, and adipokines (adiponectin and leptin)) were measured with commercial kits. We used the nephelometry technique (Behring Diagnostics, Marbury, Germany) to measure hsCRP, quantitative sandwich enzyme immunoassay technique (R&D systems, Minneapolis, Minn, USA) to determine level of IL-6, a human adiponectin RIA kit (Linco Research Lnc., Street Charles, Mo, USA) to measure serum adiponectin, and a human leptin RIA kit (Linco Research Lnc., St. Charles, Mo, USA) to measure serum leptin.

### 2.3. Statistical analysis

We performed all statistical analyses using SPSS 13.0 software and data were analyzed for normality of distribution utilizing the Kolmogorov-Smirnov test. Results are expressed as mean ± SEM for normally distributed data and as median (interquartile range) for nonparametric data. Student *t*-test was used for comparison of means between two groups and Mann-Whitney U-test for nonparametric data. We further determined correlations among all variables with Spearman rank test and performed multivariate regression analysis using Cox proportional hazard model to find factors most related to mortality within a 3-year followup. The survival analysis was conduced by the Kaplan-Meier method based on serum hsCRP and albumin level, respectively. A *P* value *<*.05 is considered as statistically significant.

## 3. RESULTS

### 3.1. Demographic and biochemical data

The mean age of the study participants was 56.3±13.6 years and the median duration of dialysis therapy was 44 months (range 6–188 months). Out of 181 patients, there were 41 deaths during the 36-month followup period. Among deceased patients, 23 patients died of infection (56.1%), 16 of cardiovascular disease (34.1%), and 2 (1.10%) of terminal cancer or accident. Table 1 shows the demographic data of deceased patients and survivors. Patients who died during followup were older and more likely to have diabetes. There was no significant difference in the duration of hemodialysis, gender, and BMI between two groups. Adequacy of dialysis, as indicated by $\text{K}\text{T}/{\text{V}}_{\text{urea}}$ and URR, is similar in the two groups. Protein intake, represented as nPCR, was significantly greater in the survivors. Deceased patients had significantly greater levels of serum hsCRP, IL-6, and lower serum albumin level. There were no significant differences in hemoglobin, total cholesterol, triglycerides, serum adiponectin, and leptin between the two groups. There was no difference in adiponectin levels between renin angiotensin inhibitors use and nonuse patients (data not shown).

Lower but not statistically significant adiponectin level was observed in diabetics (median:15.52 ug/mL, range: 4.3–88.03 ug/mL) than in nondiabetics (median 21.4 ug/mL, range: 2.43–76.45 ug/mL).

### 3.2. Correlation study

Serum hsCRP positively correlated with IL-6 (*r* = 0.62, *P* *<* .001), BMI (*r* = 0.304, *P* *<* .001), and leptin (*r* = 0.293, *P* *<* .001) but was negatively correlated with serum albumin (*r* = −0.486, *P* *<* .001) and adiponectin (*r* = −0.225, *P* *<* .001). No association between CRP and nPCR was found (*r* = −0.092, *P* = .225). IL-6 positively correlated with leptin (*r* = 0.232, *P* *<* .005) and BMI (*r* = 0.287, *P* *<* .01) but not with adiponectin (*r* = −0.115, *P* = .255) or nPCR (*r* = −0.043, *P* = .672). There was a negative correlation between leptin and adiponectin levels (*r* = −0.191, *P* *<* .05). Serum albumin correlated positively with nPCR (*r* = 0.261, *P* *<* .001) and negatively with serum hsCRP and IL-6 (*r* = −0.414, *P* *<* .001) but had no significant relationship with leptin (*r* = −0.137, *P* = .084), adiponectin (*r* = 0.024, *P* = .753), or BMI (*r* = −0.010, *P* = .181). The BMI positively correlated with serum hsCRP and leptin (*r* = 0.404, *P* *<* .001) and negatively with serum adiponectin (*r* = −0.287, *P* *<* .001).

### 3.3. Cox-regression analysis

We used the significant correlation coefficients in a Cox-regression analysis (Table 2). The results show that advanced age, serum hsCRP level, albumin level, and presence of diabetes mellitus are independent predictors for mortality in chronic hemodialysis patients.

### 3.4. Survival analysis

**Figure 1:**
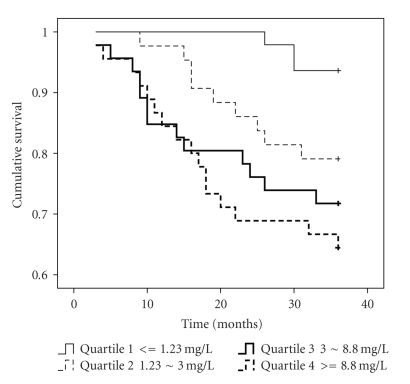
Kaplan-Meier survival curve for all-cause mortality in hemodialysis patients. We classified patients into four quartiles based on serum hsCRP levels.

The Kaplan-Meier survival curve, determined from baseline hsCRP level (Figure 1), demonstrated that patients within the highest quartile (hsCRP > 8.8 mg/L) had the highest mortality (3-year survival rate: 64.4%) in comparison with the other three quartiles (hsCRP *<* 1.23 mg/L: 93.6%, hsCRP 1.23 to 3.0 mg/L: 79.1%, hsCRP 3.1 to 8.8 mg/L: 71.7%, all *P* *<* .05). There was no significant difference between the other three quartiles. Baseline albumin level predicts survival rate and patients in the lowest group (albumin *<* 3.5 g/dL) had the lowest survival rate (63.2%) compared with the other three quartiles (albumin 3.5 to 3.7 g/dL: 81.4%, albumin 3.8 to 4.0 g/dL: 89.7%, albumin > 4.0 g/dL: 95.5%, all *P* *<* .05). The survival rate was similar between the other three quartiles.

## 4. DISCUSSION

Our results clearly demonstrate that patients with more signs of inflammation and lower albumin levels face an increased risk of all-cause mortality. Furthermore, comparison between survivors and deceased patients found that deceased patients were older and more likely to have diabetes. It has been long recognized that diabetes is an independent predictor of mortality in dialysis patients [9, 10]. However, in contrast to previous studies, we found that hypertension did not contribute to mortality. It is possible that control of blood pressure in our patients may have reduced the impact of blood pressure on overall mortality.

Previous studies have shown that CRP, a product of the inflammatory response, contributes to atherosclerosis, cardiovascular risk, and mortality in dialysis patients [11, 12]. Our study shows, as expected, that hsCPR level correlated with BMI and IL-6 levels, but inversely correlated with serum albumin and adiponectin. Moreover, hsCRP is an independent factor associated with risk of mortality in the present study. This result illustrates the critical importance of inflammation in dialysis population. As an upstream pro-inflammation marker, IL-6 promotes expression of CRP gene in the liver, and elevated level is a strong predictor of atherosclerosis and mortality in dialysis patients [13]. Although IL-6 level was lower in the survivals, our study did not support its predicting role.

The prevalence of malnutrition is as high as 34% to 60% in ESRD patients, and the impact of malnutrition on patient outcome has been investigated [14]. However, a single measurement of serum albumin is more closely related to mortality and inflammation than nutritional status in patients near the start of dialysis therapy [4]. Furthermore, hypoalbuminemia is not only presented as malnutrition, it is also the most analytical factor of graft thrombosis, arthrosclerosis, and can predict all causes and cardiovascular mortality in dialysis patients [15]. Our results show that albumin level independently predicts mortality, representing its unique role that is not necessarily related to inflammation. Among dialysis patients, many factors other than inflammation can influence serum albumin levels [16]. Thus, the relationship between inflammation and malnutrition is complex. Though serum albumin level was related with protein intake, lack of association between nPCR and inflammatory markers suggests that albumin level *per se* is not solely a marker of nutritional status in dialysis patients.

Adipose tissue stores energy and also secretes inflammatory cytokines (leptin, adiponectin, resistin, IL-6, and tumor necrotic factor alpha) that contribute to systemic inflammation. Zoccali et al. [17] performed a longitudinal study in chronic renal failure patients and found that leptin was upregulated when an infection triggered acute inflammation. They considered this adipokine as an inverse acute-phase reactant. With the positive correlation of leptin with hsCRP and IL-6, our study supports this conclusion. Previous studies have shown that plasma adiponectin level is a negative predictor of cardiovascular outcomes among patients with ESRF [18], and has inverse relationship with leptin level [19]. Recent evidence suggests that adiponectin might have an antiatherogenic property and serve as a protective molecule. In our previous study, serum leptin and adiponectin levels were elevated in hemodialysis patients, and they correlated significantly with inflammation markers [20]. In the present longitudinal observation, however, we did not find that adipokines were associated with mortality. Some previous studies found that adiponectin was an independent predictor of all-cause mortality [21, 22], but other studies have not confirmed these results [23, 24]. It is unknown whether the protective effect of adiponectin was masked by the increased cardiovascular risk among dialysis patients. Further studies are needed to clarify the role of adipokine in dialysis patients. Therapeutic interventions that modulate the effect(s) of adipokines should be able to directly test this hypothesis.

Traditional risk factors such as total cholesterol and triglyceride levels examined in this study did not demonstrate the association with inflammation as well as mortality. This result is similar to previous studies [6, 15] but does not exclude the importance of dyslipidemia in dialysis patients at all. For renal anemia, successful treatment with erythropoietin therapy can save patients from anemia-related morbidity and mortality. In the study of dialysis patient outcome, there are still some modifiable and nonmodifiable factors not included in our analysis. Therefore, we can not address any conclusion on these factors with regards on mortality. On the other hand, small patient size and short followup period are another study limitation. More participants can allow us to examine study endpoints such as comorbidity and different cause of mortality.

## 5. CONCLUSIONS

Chronic inflammation is prevalent among dialysis patients who have elevated plasma adipokines. Chronic inflammation and hypoalbuminemia, but not elevated adipokines, are related to all-cause mortality. This suggests an important role of chronic inflammation and hypoalbuminemia for the outcome of long-term hemodialysis patients.

## Figures and Tables

**Figure 2:**
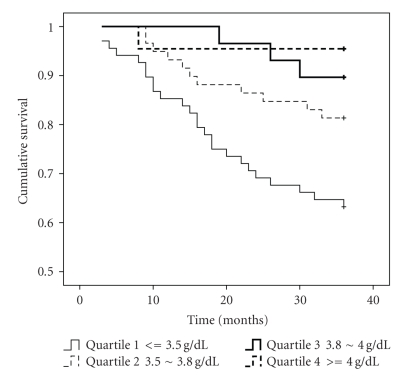
Kaplan-Meier survival curve for all-cause mortality in hemodialysis patients. We classified patients into four quartiles based on serum albumin levels.

**Table 1:** Comparison between survivors and deceased patients in our population of long-term hemodialysis patients. Data are the number of patients or the mean ± SEM for normally distributed data and median (interquartile range) for nonparametric data; BMI: body mass index; URR: urea reduction rate; nPCR: normalized protein catabolism rate.

	Death (*n* = 41)	Survival (*n* = 140)	*P value*
Age (years)	64.3±11.6	53.9±13.2	**<* 0.001*
Gender (male)	24	73	*0.470*
Diabetes mellitus	22	104	*0.012*
Hypertension	28	97	*0.904*
Duration on dialysis (months)	46.0 (19−90)	44.0 (19−76.5)	*0.803*
BMI (kg/m^2^)	23.1±4.3	22.9±3.4	*0.803*
URR (%)	0.75±0.07	0.74±0.06	*0.217*
Kt/V	1.44±0.28	1.37±0.24	*0.172*
nPCR (g/kg/day)	1.09±0.30	1.21±0.24	*0.032*
Hs-CRP (mg/L)	7.07 (2.59−13.24)	2.28 (0.98−7.84)	**<*0.001*
IL-6 (pg/mL)	6.27 (2.67−12.39)	2.83 (0.2−7.78)	*0.039*
Serum albumin (g/dL)	3.42±0.36	3.72±0.33	**<*0.001*
Leptin (ng/mL)	14.8 (7.43−38.08)	15.2 (6.48−33.85)	*0.492*
Adiponectin (ug/mL)	20.78 (10.45−34.0)	20.48 (13.48−30.37)	*0.751*
Hemoglobulin (g/dL)	9.6 (8.7−10.7)	9.8 (8.8−10.8)	*0.922*
Total cholesterol (mg/dL)	175.0±43.4	191.5±44.5	*0.077*
Triglyceride (mg/dL)	136.0 (96−262.5)	161.5 (106.80−229.8)	*0.786*

**Table 2:** Cox-regression analysis for independent predictors of mortality in hemodialysis patients.

	Wald	Relative risk	*P value*
Hs-CRP	11.731	1.046	*0.001*
Diabetes mellitus	4.868	0.467	*0.027*
Age	5.024	1.038	*0.025*
Albumin	5.565	0.274	*0.018*
